# From FAIR to FAIRS: Data security by design for the global burden of animal diseases

**DOI:** 10.1002/agj2.21017

**Published:** 2022-03-30

**Authors:** Deborah Stacey, Kenneth Wulff, Nidhip Chikhalla, Theresa Bernardo

**Affiliations:** ^1^ School of Computer Science Univ. of Guelph Guelph ON N1G 2W1 Canada; ^2^ Population Medicine, Ontario Veterinary College Univ. of Guelph Guelph ON N1G 2W1 Canada

## Abstract

Solving complex global problems involving data and data analysis can require data from both the public and private sectors. The sharing of data has traditionally been restricted to open data. To facilitate the use of both open and private data, a new data‐sharing framework has been constructed as an extension to the popular Findable‐Accessible‐Interoperable‐Reusable (FAIR) framework. The “Secure by Design” approach has been taken to define the FAIRS data‐sharing framework where S stands for Secure. A Cloud infrastructure architecture is proposed that would allow data brokers to implement FAIRS. This architecture is being constructed for the Global Burden of Animal Diseases (GBADs) to facilitate the sharing of livestock data.

## Core Ideas

- Solving complex global problems will require data from both the public and private sectors.
- Security is a primary consideration for sharing private sector data.
- Security must be a primary driver of design for systems that intend to include private data.
- To extend the FAIR data‐sharing framework to the private sector we need to add S for Secure.

**Abbreviations**
AWS: Amazon Web Services
FAIR: Findable‐Accessible‐Interoperable‐Reusable
GBADs: Global Burden of Animal Diseases
GBD: Global Burden of Disease
S3: Simple Storage Service

## 1 INTRODUCTION

### 1.1 Introduction to Global Burden of Animal Diseases

The Global Burden of Animal Diseases (GBADs) is an international initiative that will improve decision‐making for animal health by integrating data from a variety of sources and sharing tools for analysis (GBADs, 2020). It will contribute to the United Nations’ Sustainable Development Goals of zero hunger, good health and well‐being, gender equality, and responsible consumption and production (Reyers et al., 2017; United Nations, 2015). The GBADs has multiple theme groups, one of which is Informatics.

The GBADs is inspired by the Global Burden of Disease (GBD) (GBD, 2020) for humans. Started in 1990, GBD provides a comprehensive picture of what disables and kills people over time. Data on death and disability are collected and analyzed by a consortium of more than 3,600 researchers in more than 145 countries using models and other tools. By quantifying the losses at global, national, and local levels, GBD supports strategic investments in health systems to improve outcomes and reduce disparities.

While recent events in 2020 have made the world aware of the danger of global pandemics like COVID‐19 for humans, epidemics and endemic diseases of livestock threaten food and economic security. This is particularly true for regions of the world that are developing their livestock‐derived protein for consumption or trade. In June 2020, farmers in Nigeria suffered devastating losses as hundreds of thousands of pigs had to be culled in response to an epidemic of African swine fever. Animal diseases can also affect human health because some animal diseases can spread to human populations (zoonotic diseases). If that disease can subsequently be transmitted from human to human, we have the setting for another possible pandemic. Many scientists fear that the potential for future epidemics emerging from zoonotic diseases is increasing, and there are calls for increased surveillance and monitoring (Daszak et al., 2020; Lee, 2019).

Although inspired by GBD, GBADs faces additional challenges to realizing its mission. Global Burden of Disease covers one species, whereas GBADs will have to cover the breadth of species that make up the world agricultural (terrestrial and aquatic) animal domain. The GBADs also has a different philosophical approach; the data and tools of GBADs will be decentralized, Cloud‐based, and look beyond open‐data sharing to the world of private, industrial, and commercial data sharing. This last point is the driving force behind the concepts in this paper. By designing the informatics infrastructure and systems to facilitate the sharing of both open and private data, we are moving beyond the design purpose of many academia‐led projects, acknowledging that security will be one of the primary drivers of design.

### 1.2 The challenges of sharing data

There are many challenges with sharing data, regardless of whether the data are open or private or whether the participating organizations are academic, governmental, nongovernmental, or commercial entities. First, there is the problem of defining what is even meant by data sharing. To illustrate that this is not a trivial exercise, examine the definition of data sharing in Wikipedia (Wikipedia, 2020): “Data sharing is the practice of making data used for scholarly research available to other investigators.” This definition identifies the source and users of shared data as both being in academia. A more nuanced approach is taken by the Open Data Institute (ODI, 2020a) that defines data as being on a spectrum from closed (i.e., private in our terminology) to shared to open. They actively promote data sharing in the private sector. They see this as the notion of businesses sharing data within a sector, industry, network, or with other individual businesses (ODI, 2020b). In other words, they restrict data sharing to just those data that are made “open” by the private sector. Unfortunately, this is currently a very small percentage of private data, and it does not seem likely that this situation will change in the near future. There are many issues with turning some data into public entities such as privacy concerns, the dangers involved in the theft of personally identifiable information, costs involved in de‐identifying data, etc. Even if there is a desire to share data, sharing is not a simple, inexpensive, or singular activity.

For the GBADs project, we see data sharing as going beyond the sharing of open data. If we want to improve the quality, velocity, and scope of livestock data, we must establish a way to share data generated by the private sector directly from the farmers, veterinarians, food processors, and retailers. Much of the open data on livestock can take months or even years to appear. Even with the open data that are shared by major governmental or nongovernmental organizations, the time to release data can lag years behind. Often the reason for this lack of speed involves the need to process data from many separate (usually governmental) entities that are all working on different time scales and have different comfort levels about releasing data that are still being verified. A lack of standards for metadata around data quality, verification, and validation creates a situation where this year's data about livestock will not be available for 1–2 yr. In livestock research, we need to speed up our “time to science” by utilizing cutting edge computing and communications technologies such as Cloud and Edge computing, data streaming, semantic computing, serverless functions, and advanced security techniques and privacy protocols to accomplish this objective.

**Core Ideas**

- Solving complex global problems will require data from both the public and private sectors.
- Security is a primary consideration for sharing private sector data.
- Security must be a primary driver of design for systems that intend to include private data.
- To extend the FAIR data‐sharing framework to the private sector we need to add S for Secure.

The solution to this data‐sharing challenge for GBADs is applicable beyond this project. There is often a disconnect between academic researchers and private organizations over the proper way to share data. The negotiations on protocols can take a great deal of time and effort. Nonetheless, the agreed data transfer and storage mechanisms then often fall short of ensuring the security and privacy of the data.

In approaching this challenge, we will first examine the technological issues with the sharing of data across the spectrum of identities (from totally open to shared‐with‐conditions to restricted, private data), and we will limit our scope to data sharing in the Cloud. This is not truly a limitation as we will demonstrate that Cloud data sharing can be secure, stable, fast, and can facilitate our vision of a decentralized, nonhierarchical organization of data, tools, and models to help serve people, animals, and society. In the near future, there will be an expansion of the number and size of livestock datasets available from many different shareholders, including the individual farmer or pastoralist, agri‐food corporations, academic researchers, and governmental and inter‐governmental agencies, thus making this proposed technology even more necessary.

### 1.3 Misconceptions about data security in the Cloud

Cloud computing is the provisioning of computing and digital storage infrastructure and the delivery of digital services over the Internet. Infrastructure and services on modern Cloud platforms such as Amazon Web Services (AWS), Microsoft Azure, and Google Cloud Platform, include computer servers, data storage, networking, software, analytics, monitoring, and applications such as streaming video and machine learning. To address the usage of Cloud computing, we will start by challenging some popular misconceptions about data security in the Cloud. Many people in both the private and public sectors believe that the use of on‐premise computer server technology is inherently more secure than data storage in the Cloud (Shakya, 2019). While there are numerous instances of the hacking of on‐premise servers, many are quick to point to the instances of data breaches in the Cloud to back up their opinions. Let us start by examining three recent instances of significant data breaches.

### 1.4 Data breaches

In early 2020, an AWS Simple Storage Service (S3) bucket (a data‐storage structure) owned by Data Deposit Box, a Canada‐based public company that offers secure Cloud backup storage service, was found to be completely unsecured and unencrypted. Data Deposit Box had approximately 350,000 users spread over 84 countries, who could continuously back up their data through Data Deposit Box's services. A research team led by cybersecurity analysts discovered a serious breach while monitoring Cloud resources over the Internet. Using just a web browser, anyone could query databases owned by Data Deposit Box. Because S3 buckets are secure by default (i.e., private and not accessible by the public), the insecurity of these buckets was caused by misconfiguration of the Cloud data storage by an employee of Data Deposit Box. The breach was secured within 7 d of notification (Coble, 2020a).

Around the same time, Microsoft accidentally exposed anonymized user analytics data collected from customer support interactions. This exposure of about 250 million data entries was discovered by an independent security researcher. The problem was caused by misconfiguration of Microsoft Azure's security policies. The breach was secured within 24 h (Ikeda, 2020).

The largest data breach in Canadian history occurred in October 2019, when the medical data of over 15 million Canadians were exposed by LifeLabs. The data exposed included lab test results, health card numbers, and personally identifiable information such as names, dates of birth, home addresses, email addresses, login IDs, and passwords. It is alleged that the data in question were stored on unsecured servers and that the data were not encrypted. It has also been claimed that the network security personnel responsible for securing the data were not properly trained and that there was not an adequate number of staff (Coble, 2020b), but this data breach was not in the Cloud. LifeLabs's own systems were hacked and its data ransomed, revealing a shocking level of insecurity by a company entrusted with medical data.

What lessons can be learned from this brief look at recent data breaches? The data breaches in the Cloud were all caused by misconfiguration of Cloud resources by data owners and not from inherent insecurities in the Cloud platforms. The systems were not hacked; the data were exposed through the actions of the data owners. A review of security measures available on Cloud platforms reveals that these misconfiguration problems are relatively straightforward to overcome by better staff training (Morrow et al., 2019), use of security tools provided by the platforms, and regular monitoring and audits that can expose and correct security issues quickly and decisively.

The differentiation of responsibility for security in the Cloud is commonly referred to as Security “of” the Cloud versus Security “in” the Cloud. In the first case, the Cloud provider operates, manages, and controls the components from the host operating system and virtualization layer down to the physical security of the facilities in which the service operates. The Cloud user assumes responsibility and management of the operating systems on their computation resources as well as application software and configuration security entities such as firewalls (AWS, 2020). In this model, the Cloud platform does much of the work to ensure security.

On‐premise servers, networks, software, and staffing are far more likely to have many more security issues than Cloud‐based solutions. Also, the cost in time and effort for securing on‐premise data centers is much greater relative to purchasing these services from public Cloud platforms because of economies of scale and domain expertise. Public Cloud platforms have infrastructure security at a scale that is attainable by very few organizations, businesses, or even governments. Security applied to their hardware, networks, software, staffing, and geolocation is superior to any public‐facing infrastructure currently available.

## 2 MATERIALS AND METHODS

### 2.1 “Secure by Design’’ to facilitate data sharing

How do you balance data security and privacy (for data owners) with ease of use and timely, continuous availability (for researchers)? How can open data and private data exist in the same system and provide ease of use and availability for one and security and privacy for the other? We will develop our philosophy of facilitated multi‐spectral data sharing (open–shared–private) by extending an existing set of guidelines for data sharing. We will extend the data‐sharing framework Findable‐Accessible‐Interoperable‐Reusable (FAIR) to FAIRS where S stands for Security.

### 2.2 From FAIR to FAIRS

In 2016, the paper “The FAIR Guiding Principles for scientific data management and stewardship” set out principles for data stewardship in the life sciences (Wilkinson et al., 2016). The FAIR Data Principles put emphasis on machine‐to‐machine data communication, which is exactly what the GBADs initiative needs. The principles state that data must be:

- **F**indable: have rich metadata resources registered or indexed in a searchable resource
- **A**ccessible: use standard protocols for accessing and retrieving data with authentication/authorization in place, if necessary
- **I**nteroperable: use a formal, shared language for knowledge representation along with standard vocabularies
- **R**eusable: use clear and accessible data usage license(s) and store detailed data provenance

While the authors of the FAIR paper claim that their framework does include private data, it does not explicitly address the security aspect of data sharing. Because the FAIR data principles have gained a degree of general acceptance in the academic community (Boeckhout et al., 2018), we propose to extend these principles by transforming FAIR to FAIRS by adding the principle: To be Secure.

### 2.3 S is for secure

We have adopted the FAIR format for explaining our additional principle and propose subprinciples S1 to S6.

#### 2.3.1 Secure

Ensure that data are secure by default with appropriate access permissions, authorization, views, monitoring, and encryption using standard cybersecurity protocols and best practices for automation and staff education and training.

#### 2.3.2 S1: All data are secure by default

Security has become ubiquitous in everyday computing, and it is even more important when it comes to storing sensitive data. It is necessary for security to be the underlying thread that binds other FAIRS principles together. Many organizations are beginning to ask if their data platforms or systems are designed to be secure by default. Recent and ongoing data and privacy breaches have brought this into focus. A FAIRS data platform should allow:

- **F**inding and **A**ccessing its data in a safe and **S**ecure manner
- **I**nteroperability or integration with other systems designed to guarantee the **S**ecurity and privacy of the data
- **R**eusability of the data with **S**ecurity in mind

#### 2.3.3 S2: All data have appropriate access permissions

It is important to grant the necessary access permissions so that users and applications have just the right authorization to access the specific datasets they require to do their work. The Principle of Least Privilege (Saltzer & Schroeder, 1975) states that a subject should be given only those privileges needed to complete its task. If a subject does not need an access right, the subject should not have that right. Function(s), as opposed to identity, should control rights assignment.

#### 2.3.4 S3: Secure management of user identities and authorizations

Users can be categorized into groups based on their functions or roles and consequently given the needed authentication and authorization to perform their duties. Groups with defined privileges are much safer than treating each user separately. This concept is already a part of the identity and access management systems of the major public Cloud platforms. When duties or functions change, previous roles (permissions) are revoked, and new roles assigned (as part of a group). This sub‐principle emphasizes the need to organize functions and roles for users in a manner that is straightforward to learn and manage for Information Technology staff.

#### 2.3.5 S4: Access methods manage views of data for identified users, roles, and functions

A “view” can be defined as part of privilege management. It will allow access to parts of the data collected (no direct querying of the data) or access to only parts of a data record (masking). Access to views is based on privilege. These privileges can be carefully curated and monitored by the data owner. A view can be used to present aggregated and de‐identified data from one or more data collection repositories (virtual data sets).

Why are we looking at re‐purposing the database concept of views? A view can limit the degree of exposure of the underlying data and hide the complexity of the data. Views provide extra security because users are only allowed to see virtual data in a way that is controlled by the data owner.

#### 2.3.6 S5: Encryption is used when necessary for nonopen data

After establishing data threats and risks, encryption can be used to protect the data both in transit or at rest. Data in transit can be protected using Secure Sockets Layer or client‐side encryption, and data at rest can be protected using server‐side encryption and database encryption. Encryption does decrease the speed and ease of sharing, but for personally identifiable information and other data, there is no substitute for this type of protection in case a breach occurs.

#### 2.3.7 S6: Machine‐to‐machine aspects of security versus accessibility

The FAIR framework has promoted changing the assumption that data are consumed by people and recognizes that most data are consumed by machines through application programming interfaces (APIs), file transfers, etc. Machine‐to‐machine transmission can be achieved by ensuring that both the source and destination endpoints are secure by using the most up‐to‐date security protocols. Public and private data spheres can exist in parallel in the same Cloud‐based system. User/API experiences will be different: public users do not need to self‐identify, while users of private data will have to go through authentication and authorization. The underlying infrastructure for data storage and computation can be separately provisioned and a gateway to the system can direct queries to the appropriate “side” of the system. A “load balancer,” like AWS Elastic Load Balancing, Azure Load Balancer, or Google Load Balancing, can direct Internet traffic (i.e., requests for data) to targets within a system's Cloud infrastructure based on the content of the request. In this manner, the requests for data operations will contain the information needed to assign them to the appropriate part of the data spectrum. A user of open data need not identify or authenticate, but a user of private data will have to do both.

**FIGURE 1:**
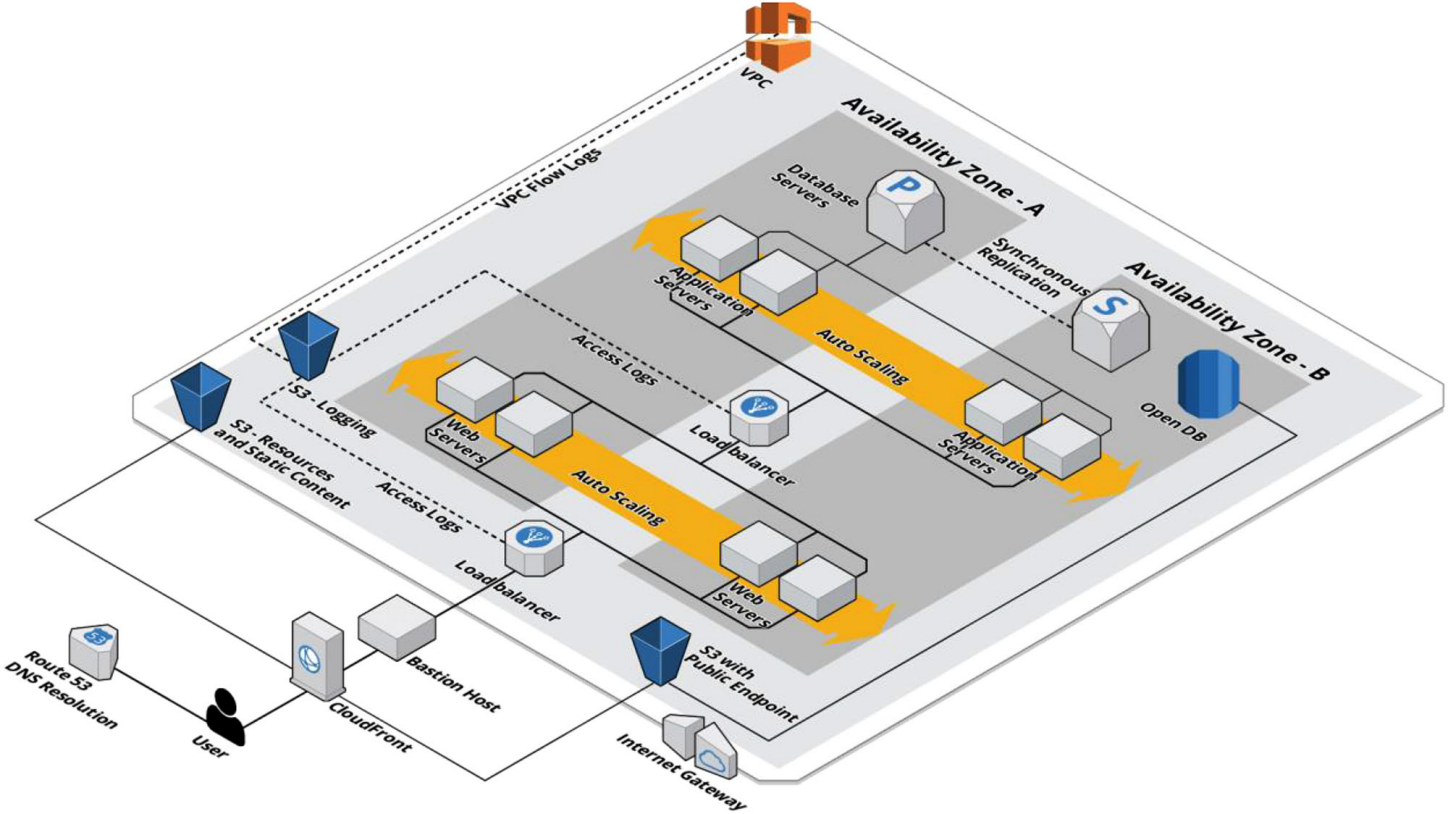
A secure and modular Cloud architecture for sharing open and private data

**FIGURE 2:**
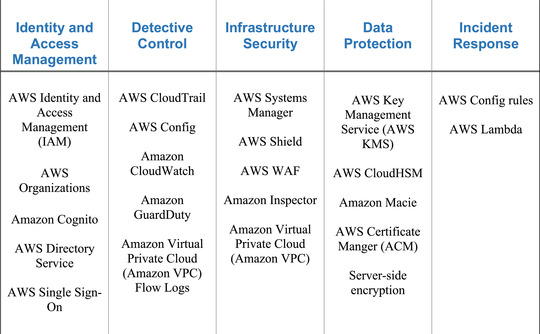
Categories of Cloud services needed to implement the full architecture

The Cloud architecture in Figure 1 illustrates an AWS Cloud infrastructure that facilitates all of the FAIRS principles. This architecture is dynamically expandable and constructed so that even more security mechanisms from the Cloud platform can easily be integrated. While not the simplest of architectures, it is also not so complex as to preclude implementation by most organizations. This architecture secures data transfers, data storage based on our data spectrum (open–shared–private), user identity and access management, and Cloud security and monitoring. Figure 2 breaks the services used in this infrastructure into five categories: identity and access management, detective control, infrastructure security, data protection, and incident response. All the services in this table come from the AWS Cloud platform. This is only used as an example platform. The architecture can readily be adapted to other Cloud platforms such as Microsoft Azure and Google Cloud Platform by mapping the services in Figure 2 to the equivalent ones in the platform of choice. Further research on this architecture will concentrate on developing mappings to these other Cloud platforms and formally defining the modularity characteristics of this architecture.

## 3 RESULTS AND DISCUSSION

Data sharing across the spectrum of data modalities (open, shared, private) is attainable in the Cloud by extending the FAIR data sharing principles to FAIRS (FAIR with Security). For too long, the data‐sharing conversation in academia has centered on the sharing of open data and the promise of convincing industry to share their data in some form. While that is a laudable goal, it is not always realistic for some types of data.

Our proposal is to design a data‐sharing system with security in mind. Data security is attainable in the Cloud because of the security and stability of the underlying Cloud platform infrastructure combined with a known set of best practices as outlined by many agencies (e.g., vendors, Cloud providers, security organizations). Proper training of systems staff can also help alleviate the major factor in Cloud data insecurity—misconfiguration. Our principles and observations have allowed us to generate a proposed Cloud architecture that can serve any organization that needs to share data in all of its forms, including private data. This architecture is probably best implemented by a trusted data broker. We envision GBADs as one such broker, but many universities and research institutions could also function as brokers to link research with industry for the benefit of all.

In addition to adjusting FAIR to FAIRS in our system design, we will also use data governance principles based on FAIRS to achieve the goals that will allow GBADs to be a platform for the sharing of data, tools, models, and other products for decision‐making in the domain of livestock systems. The following goals are derived from those articulated in the book, “Democratizing our Data: A Manifesto” by Julia Lane (Lane, 2020). They will be accomplished using FAIRS data‐governance mechanisms, such as meta‐data, provenance, user roles, etc., and extending these using semantic tools such as graph databases, ontologies, and knowledge graphs. This form of data governance will also be used to promote “semantic interoperability” between datasets that goes beyond the “data interoperability” proposed by traditional FAIR. We envision that GBADs semantic interoperability will align with that of the European Commission's Interoperability Framework (EIF, 2021), which is defined as, “Semantic interoperability ensures that the precise format and meaning of exchanged data and information is preserved and understood throughout exchanges between parties, in other words ‘what is sent is what is understood’.” We anticipate that our FAIRS principles and the platforms developed from it will facilitate research towards interoperability across livestock datasets. In the near future, the GBADs Cloud‐base Informatics platform will use FAIRS to share:

- Data that can be used in a **timely** manner for decision making.
- Data that have been analyzed for **quality,** and these metrics are available as part of the meta‐data.
- As **complete** a data collection as possible for all its stakeholders. This will include data that GBADs redirects from other organizations and data that GBADs stores for associated projects and partners.
- Data that are **relevant** to the modelling, decision support, and other purposes important to the aims of GBADs and its users.
- Data that are easily **accessible** to appropriate users (both machine and human).
- Data that are **interpretable**—the semantics to be encoded in meta‐data and other semantic systems.
- Access to technology and products that are **innovative** and advance the state‐of‐the‐art.
- A system that allows for the **customization** of information through mechanisms and processing that adjusts the granularity of the data to the user's needs.

## AUTHOR CONTRIBUTIONS

Deborah Stacey: Conceptualization; Formal analysis; Investigation; Methodology; Project administration; Writing – original draft; Writing – review & editing. Kenneth Wulff: Writing – original draft. Nidhip Chikhalla: Writing – original draft. Theresa Bernardo: Writing – original draft; Writing – review & editing.

## CONFLICT OF INTEREST

The authors declare no conflicts of interest.

## References

[agj221017-bib-0001] Amazon Warehouse Services (AWS). (2020). Shared Responsibility Model . Amazon. https://aws.amazon.com/compliance/shared‐responsibility‐model/

[agj221017-bib-0002] Boeckhout, M. , Zielhuis, G. A. , & Bredenoord, A. L. (2018). The FAIR guiding principles for data stewardship: Fair enough? European Journal of Human Genetics, 26, 931–936. 29777206 10.1038/s41431-018-0160-0 PMC6018669

[agj221017-bib-0003] Coble, S. (2020a). Data deposit box exposes PII of 270K users . Infosecurity Group. https://www.infosecurity‐magazine.com/news/data‐deposit‐box‐exposes‐pi‐of/

[agj221017-bib-0004] Coble, S. (2020b). Lawsuit filed against LifeLabs over data breach . Infosecurity Group. https://www.infosecurity‐magazine.com/news/lawsuit‐filed‐against‐lifelabs/

[agj221017-bib-0005] Daszak, P. , Olival, K. J. , & Li, H. (2020). A strategy to prevent future epidemics similar to the 2019‐nCoV outbreak. Biosafety and Health, 2(1), 6–8. 32562482 10.1016/j.bsheal.2020.01.003 PMC7144510

[agj221017-bib-0006] European Interoperability Framework (EIF) (2021). Interoperability layer 5: Semantic interoperability . https://joinup.ec.europa.eu/collection/nifo‐national‐interoperability‐framework‐observatory/solution/eif‐toolbox/interoperability‐layer‐5‐semantic‐interoperability

[agj221017-bib-0007] Global Burden of Animal Diseases (GBADs) (2020). https://animalhealthmetrics.org/ 10.20506/rst.43.3519 39222110

[agj221017-bib-0008] Global Burden of Disease (GBD) (2020). http://www.healthdata.org/gbd/about

[agj221017-bib-0009] Ikeda, S. (2020). 250 million Microsoft customer service records exposed; Exactly how bad was it? CPO Magazine. https://www.cpomagazine.com/cyber‐security/250‐million‐microsoft‐customer‐service‐records‐exposed‐exactly‐how‐bad‐was‐it/

[agj221017-bib-0010] Lane, J. (2020). Democratizing our data: A manifesto. MIT Press.

[agj221017-bib-0011] Lee, K. (2019). Future management strategies for zoonoses based on one health. Journal of Agricultural Medicine and Community Health, 44(1), 39–42.

[agj221017-bib-0012] Morrow, T. , LaPlana, V. , Faatz, D. , & Hueca, A. (2019). Cloud security best practices derived from mission thread analysis (Technical Report CMU/SEI‐2019‐TR‐003 CERT Division).

[agj221017-bib-0013] Open Data Institute (ODI) (2020a). https://theodi.org/

[agj221017-bib-0014] Open Data Institute (ODI) (2020b). The value of data sharing in the private sector . https://theodi.org/project/the‐value‐of‐data‐sharing‐in‐the‐private‐sector/

[agj221017-bib-0015] Reyers, B. , Shrubsole, K. , & Power, E. (2017). Essential variables help to focus Sustainable Development Goals monitoring. Current Opinion in Environmental Sustainability, 26–27, 97–105. 10.1016/j.cosust.2017.05.003

[agj221017-bib-0016] Saltzer, J. H. , & Schroeder, M. D. (1975). The protection of information in computer systems. Proceedings of the IEEE, 63(9), 1278–1308.

[agj221017-bib-0017] Shakya, S. (2019). An efficient security framework for data migration in a cloud computing environment. Journal of Artificial Intelligence and Capsule Networks, 1(1), 45–53.

[agj221017-bib-0018] United Nations (2015). Transforming our world: The 2030 agenda for sustainable development. United Nations General Assembly, Seventieth Session.

[agj221017-bib-0019] Wikipedia (2020). Data sharing . https://en.wikipedia.org/wiki/Data_sharing

[agj221017-bib-0020] Wilkinson, M. D. , Dumontier, M. , Aalbersberg, I. J. , Appleton, G. , Axton, M. , Baak, A. , Blomberg, N. , Boiten, J. W. , da Silva Santos, L. B. , Bourne, P. E. , Bouwman, J. , Brookes, A. J. , Clark, T. , Crosas, M. , Dillo, I. , Dumon, O. , Edmunds, S. , Evelo, C. T. , Finkers, R. , …, & Mons, B. (2016). The FAIR guiding principles for scientific data management and stewardship. Scientific Data, 3, 160018. 10.1038/sdata.2016.18 26978244 PMC4792175

